# Prognostic value of heart failure hospitalization in transthyretin cardiac amyloidosis: an international cohort study

**DOI:** 10.1093/eschf/xvaf013

**Published:** 2026-01-08

**Authors:** Dorien Laenens, Philippe Debonnaire, Maarten A J De Smet, Fausto Pinto, Dulce Brito, Steven Droogmans, Frederik H Verbrugge, Erwan Donal, Nico Van de Veire, Philippe Bertrand, Ching Hui Sia, Arnold C T Ng, Takeru Nabeta, Nicole Sturkenboom, Idit Yedidya, Ruxandra Jurcut, Jeroen J Bax, Nina Ajmone Marsan

**Affiliations:** Department of Cardiology, Leiden University Medical Center, Albinusdreef 2, Leiden 2333 ZA, Netherlands; Department of Cardiology, Sint Maarten Hospital, Mechelen, Belgium; Department of Cardiology, Sint Jan Hospital, Bruges, Belgium; Department of Cardiology, Leiden University Medical Center, Albinusdreef 2, Leiden 2333 ZA, Netherlands; Department of Cardiology, Sint Jan Hospital, Bruges, Belgium; Department of Cardiology, ULSSM, CAML, CCUL@RISE, Faculdade de Medicina, Universidade de Lisboa, Lisboa, Portugal; Department of Cardiology, ULSSM, CAML, CCUL@RISE, Faculdade de Medicina, Universidade de Lisboa, Lisboa, Portugal; Faculty of Medicine and Life Sciences, Vrije Universiteit Brussel, Brussels, Belgium; Department of Cardiology, University Hospital Brussels, Brussels, Belgium; Faculty of Medicine and Life Sciences, Vrije Universiteit Brussel, Brussels, Belgium; Department of Cardiology, University Hospital Brussels, Brussels, Belgium; Department of Cardiology, CHU de Rennes, Université de Rennes, Rennes, France; Faculty of Medicine and Life Sciences, Vrije Universiteit Brussel, Brussels, Belgium; Department of Cardiology, AZ Maria Middelares, Gent, Belgium; Department of Cardiology, Hospital Oost-Limburg, Genk, Belgium; Department of Cardiology, National University Heart Centre, Singapore, Singapore; Department of Cardiology, University of New South Wales, Sydney, New South Wales, Australia; Department of Cardiology, Leiden University Medical Center, Albinusdreef 2, Leiden 2333 ZA, Netherlands; Department of Cardiology, Kitasato University School of Medicine, Sagamihara, Japan; Department of Cardiology, University Hospital Antwerp, Antwerp, Belgium; Department of Cardiology, Rabin Medical Center, Petah Tikva, Israel; Department of Cardiology, Faculty of Medicine, Tel Aviv University, Tel Aviv, Israel; Institute of Cardiovascular Diseases, Bucharest, Romania; Department of Cardiology, Leiden University Medical Center, Albinusdreef 2, Leiden 2333 ZA, Netherlands; Department of Cardiology, Turku Heart Center, University of Turku and Turku University Hospital, Turku, Finland; Department of Cardiology, Leiden University Medical Center, Albinusdreef 2, Leiden 2333 ZA, Netherlands

**Keywords:** Transthyretin cardiac amyloidosis, Heart failure hospitalization, Risk stratification

## Abstract

**Introduction:**

Data on the impact of a heart failure hospitalization (HFH) on outcome in patients with transthyretin cardiac amyloidosis (TTR-CA) are scarce, although it remains a frequent adverse event. To assess the characteristics of patients with HFH in a real-world TTR-CA population, the occurrence of HFH, and the prognosis thereafter.

**Methods:**

Data were collected from a multicentre TTR-CA registry and patients were dichotomized according to the occurrence of at least one HFH. Landmark analysis at the 1-year follow-up and Cox regression analysis with HFH as a time-dependent covariate were performed to assess the impact of HFH on all-cause mortality.

**Results:**

Overall, 654 patients were included [median age 78 (64, 83) years, 70.5% male, 70.6% wild type]. During a median follow-up of 24 (11–45) months, 141 (22%) patients experienced at least one HFH and 170 (26%) patients died. Patients with a HFH were older (82 vs 76 years, *P* < .001), had more wild-type TTR-CA [126 (89.4%) vs 336 (65.5%), *P* < .001], were more symptomatic [New York Heart Association Class II–IV 119 (86.9%) vs 279 (62%), *P* < .001], had higher National Amyloidosis Centre (NAC) disease stage, were less treated with disease-modifying therapy [45 (31.9%) vs 247 (47.4%), *P* = .001], had more co-morbidities and showed signs of more advanced disease by echocardiography. At the 1-year time point, patients with HFH had significant worse overall survival (log-rank χ² 37.673, *P* < .001). At the univariable (HR 7.71, 95%CI 5.50, 10.82; *P* < .001) and multivariable analyses, HFH was associated with all-cause mortality and showed incremental value on top of clinical variables, biomarkers [estimated glomerular filtration rate in Model 1 (χ² 97.3; *P* < .001) and NAC disease stage in Model 2 (χ² 78.8; *P* < .001)] and echocardiographic parameters (left ventricular mass index + stroke volume index + significant valvular lesion in Model 3 (χ² 60.3; *P* < .001) and including *E*/*e*′ in Model 4 (χ² 43.4; *P* < .001)).

**Conclusion:**

HFH is independently associated with all-cause mortality in patients with TTR-CA and has incremental value on top of established risk models.

## Graphical Abstract

**Graphical Abstract:**
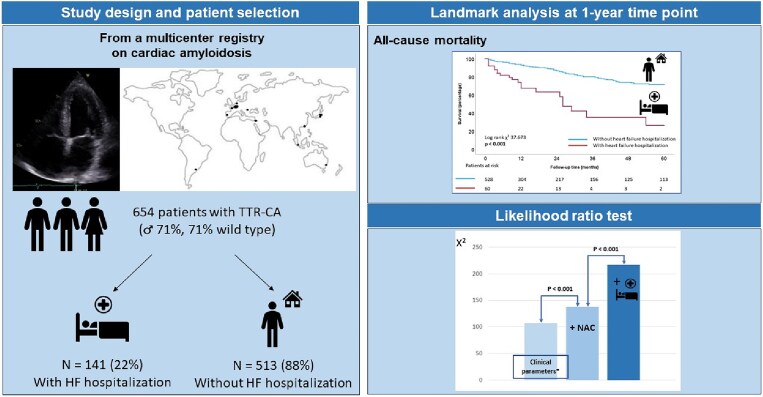
*Clinical parameters: age, transthyretin phenotype, New York Heart Association Class, presence of polyneuropathy, atrial fibrillation, disease-modifying treatment. HF, heart failure; NAC, National Amyloidosis Centre; TTR-CA, transthyretin cardiac amyloidosis.

## Introduction

Transthyretin cardiac amyloidosis (TTR-CA) is a cause of infiltrative cardiomyopathy, due to the deposition of transthyretin amyloid fibrils in the heart, which is characterized by poor prognosis. Once thought to be rare, this cardiomyopathy is increasingly diagnosed thanks to the significant advances in cardiac imaging but also to the increased awareness of the disease driven by the introduction of new disease-modifying therapies.^1–3^ Risk stratification of these patients has become therefore essential to guide clinical care, especially in the era of these expensive treatment options, which seem most efficient when started early in the disease process.^1,2^ So far, risk stratification has been predominantly based on biomarker assessment^4,5^ and echocardiographic parameters.^6^

The occurrence of a heart failure hospitalization (HFH) is generally considered harmful and a HFH is therefore included as an endpoint in many clinical HF trials.^7–10^ In patients with chronic HF, the risk of all-cause death is the highest in the first month after HFH.^11^ Also in the I-PRESERVE-trial, patients with HF and preserved ejection fraction had a significantly higher adjusted risk of mortality after the occurrence of a HFH.^12^ However, patients with hypertrophic or restrictive cardiomyopathies, and thus patients with TTR-CA, were excluded from these studies.

The aim of the current study was to assess the risk of occurrence and characteristics of a HFH, and the prognosis thereafter in a real-world cohort of patients with TTR-CA. Furthermore, the potential additive prognostic value of the occurrence of HFH over established risk factors in TTR-CA was evaluated.

## Methods

### Study population

Patients with TTR-CA were selected from a multicentre CA registry including 17 centres (Supplementary Table S1). Patients with AL amyloidosis, AA amyloidosis, unknown amyloid phenotype or missing follow-up data were excluded (Supplementary Fig. S1). Transthyretin cardiac amyloidosis was diagnosed on tissue specimens of endomyocardial biopsy, or after 2016, non-invasively by integrating the use of imaging (including bone scintigraphy and cardiac magnetic resonance imaging) and laboratory tests (to exclude the presence of monoclonal proteins) as recommended.^13^ Particularly, for patients with a TTR gene mutation, cardiac amyloidosis was defined by the presence of left ventricular (LV) hypertrophy on imaging (echocardiography or cardiac magnetic resonance imaging), not explained by abnormal loading conditions.

Demographic, clinical, laboratory, and electrocardiographic data were collected from the patient’s electronic health records of the first visit at the referral centre. National Amyloidosis Centre (NAC) disease stage was defined according to the previously defined cut-off of 45 ml/min for estimated glomerular filtration rate (eGFR) and 3000 ng/l for N-terminal pro-B-type natriuretic peptide.^5^ The study complied with the Declaration of Helsinki and the ethical committee from the participating centres waived the need for written informed consent due to the retrospective design of the study.

### Outcome data

The endpoint of this study was defined as the occurrence of all-cause mortality. In addition, the occurrence of the first HFH during follow-up was collected. HFH was defined as at least an overnight hospital stay due to typical signs of HF requiring intravenous diuretic therapy. Follow-up data were collected from electronic health records or governmental death registry database.

### Transthoracic echocardiography

All patients underwent standard two-dimensional transthoracic echocardiography at diagnosis. The images were digitally stored for offline analysis. Two-dimensional, colour, spectral continuous- and pulsed-wave Doppler images were obtained from the parasternal, apical and subcostal views. From the parasternal long-axis view, LV and left atrial dimensions were measured as recommended.^14^ Left ventricular mass index was calculated by the formula of Devereux and indexed for body surface area.^15^ From the apical four- and two-chamber view, LV volumes were measured and LV ejection fraction was calculated with the Simpson’s biplane method.^14^ Stroke volume was calculated and indexed for body surface area. Left ventricular global longitudinal strain was measured as recommended, when it was feasible, and expressed as absolute value.^16^ Tissue Doppler imaging at the medial and lateral mitral valve annulus level was used to measure e’, which was averaged to calculate *E*/*e*′, as a marker of LV filling pressures.^17^ Tricuspid annular plane systolic excursion was measured on the M-mode of the lateral tricuspid annulus to estimate the right ventricular systolic function.^14^ A multiparametric approach was used to assess the severity of valvular heart disease.^18,19^ The presence of ≥moderate mitral regurgitation, ≥moderate tricuspid regurgitation, ≥moderate aortic regurgitation or ≥moderate aortic stenosis (aortic valve area ≤1.5 cm²) was considered a significant valvular lesion.

### Statistical analysis

Normally distributed variables were described as mean ± standard deviation while non-normally distributed variables were described as median (interquartile range). Categorical variables were described as number with percentages. Continuous variables were compared using the independent Student *t*-test when normally distributed and with the Mann–Whitney *U* test when not normally distributed. Chi-square test was used to compare categorical variables.

To assess the influence of the occurrence of a first HFH on all-cause mortality, a landmark analysis was performed at the 1-year time point. Therefore, patients who had a HFH in the first year of follow-up were compared with those without a HFH in the first year of follow-up. Patients who died in the first year of follow-up were excluded. The analysis then started on the 366th day of follow-up. Log-rank test was used to compare the groups.

To assess the association of variables with all-cause mortality, univariable Cox regression analysis was performed. The HFH was entered as a time-dependent covariate starting from baseline on day 0. Univariable analysis was also performed in the different NAC disease stage groups and in subgroup analysis based on disease-modifying TTR treatment. Multivariable Cox regression analysis was constructed with variables associated with an increased risk of all-cause mortality in the univariable analysis (*P* < .050 and the highest Wald test statistic), taking into account the widely applied rule of 10 events per variable. Four multivariable models were constructed. Models 1 and 2 included clinical variables with biomarkers: eGFR or NAC disease stage respectively. Models 3 and 4 included the clinical variables significant in Models 1 and 2 (*P* < .050), combined with the echocardiographic variables. Stroke volume index was preferred as marker of LV systolic function over LV ejection fraction because of its previously demonstrated independent prognostic value,^6^ and over LV global longitudinal strain because of the higher missing values of this parameter. Nevertheless an additional multivariable model was tested including LV global longitudinal strain instead of stroke volume index (Supplementary Table S3) and *E*/*e*′, to show the value of these echocardiographic measures. Similar multivariable models were constructed in subgroup analysis according to disease-modifying treatment. In addition, the incremental prognostic value of the occurrence of a HFH over the conventional prognostic factors was assessed with a likelihood ratio χ² test for nested models.

## Results

### Baseline patient characteristics according to the occurrence of HFH

After applying the exclusion and inclusion criteria, 654 patients with TTR-CA were included in the study. Median age was 78 (64, 83) years, 461 (70.5%) patients were male and 462 (70.6%) had wild-type TTR phenotype. 287 (44%) were treated with disease-modifying TTR treatment (97% tafamidis, 3% TTR silencers). During a median follow-up of 24 months (10, 46), 141 (21.6%) patients encountered at least one HFH. The baseline characteristics of the total patients, divided according to the occurrence of a HFH, are presented in *Table 1*. Patients who experienced a HFH were older (*P* < .001), more symptomatic according to New York Heart Association class (*P* < .001) and had more cardiovascular co-morbidities. These patients had less often neurologic symptoms like polyneuropathy and autonomic dysfunction. The use of HF therapies like beta-blockers and mineralocorticoid receptor antagonists as well as loop diuretic use was higher in patients who experienced a HFH, while the treatment with disease-modifying TTR treatment was less frequent in patients who experienced a HFH. Due to the higher prevalence of atrial fibrillation, the use of oral anticoagulant therapy was also higher in these patients. Biomarkers were significantly more elevated in patients with HFH, reflected in a higher NAC disease stage. Electrocardiogram (ECG) characteristics showed a higher prevalence of conduction disorders in patients with a HFH, especially first degree atrioventricular block and more prolonged QRS duration. Baseline echocardiographic characteristics are presented in *Table 2*. By echocardiography, patients with a HFH exhibited a more advanced disease phenotype, with more increased LV wall thickness, more impaired LV systolic function, as by stroke volume index, LV ejection fraction and LV global longitudinal strain, and LV diastolic function (higher *E*/*e*′ and a larger left atrial diameter). Furthermore, the right ventricular function, measured by the tricuspid annular plane systolic excursion, was also more impaired in patients with HFH, who also showed higher prevalence of significant valvular lesions.

**Table 1:** Baseline clinical characteristics of the study population, further divided according to the occurrence of heart failure hospitalization

Variable	Overall (*N* = 654)	With heart failure hospitalization (*N* = 141)	Without heart failure hospitalization (*N* = 513)	*P* value
Age, years	78 (64.3, 83.3)	81.7 (76.0, 86.0)	76.0 (57.5, 82.0)	**<**.**001**
Male sex, *N* (%)	461 (70.5%)	106 (75.2%)	355 (69.2%)	.168
TTR phenotype				**<**.**001**
hereditary	192 (29.4%)	15 (10.6%)	177 (34.5%)	
wild type	462 (70.6%)	126 (89.4%)	336 (65.5%)	
BMI, kg/m²	24.9 ± 4.7	25.1 ± 4.1	24.9 ± 4.9	.568
NYHA class				**<**.**001**
I	189 (32.2%)	18 (13.1%)	171 (38.0%)	
II	265 (45.1%)	54 (39.4%)	211 (46.9%)	
III	115 (19.6%)	52 (38.0%)	63 (14.0%)	
IV	18 (3.1%)	13 (9.5%)	5 (1.1%)	
Cardiovascular risk factors and co-morbidities				
Arterial hypertension, *N* (%)	333 (51.2%)	82 (58.2%)	251 (49.2%)	.060
Dyslipidaemia, *N* (%)	284 (43.8%)	70 (50.7%)	214 (41.9%)	.063
Diabetes mellitus, *N* (%)	104 (16.0%)	29 (20.6%)	75 (14.7%)	.091
Current smoker, *N* (%)	33 (5.7%)	3 (2.4%)	30 (6.6%)	.071
Coronary artery disease, *N* (%)	146 (22.3%)	45 (31.9%)	101 (19.7%)	.**002**
Atrial fibrillation, *N* (%)	261 (46.2%)	77 (66.4%)	184 (41.0%)	**<**.**001**
Pacemaker, *N* (%)	102 (20.0%)	28 (27.7%)	74 (18.0%)	.**029**
Previous stroke, *N* (%)	**74 (14.3%)**	**22 (21.6%)**	**52 (12.6%)**	.**020**
Asthma or chronic obstructive pulmonary disease, *N* (%)	**32 (6.6%)**	**13 (14.4%)**	**19 (4.8%)**	**<**.**001**
Neurologic symptoms				
Carpal tunnel syndrome, *N* (%)	214 (32.8%)	52 (36.9%)	162 (31.6%)	.241
Polyneuropathy, *N* (%)	186 (28.5%)	24 (17.0%)	162 (31.6%)	**<**.**001**
Autonomic dysfunction, *N* (%)	153 (25.4%)	20 (16.8%)	133 (27.5%)	.**017**
Lumbar spinal stenosis, *N* (%)	118 (19.7%)	26 (22.4%)	92 (19.0%)	.413
Medication use				
Beta blocker, *N* (%)	282 (43.2%)	72 (51.1%)	210 (41.0%)	.**033**
ACE-i/ARB, *N* (%)	265 (40.6%)	64 (45.4%)	201 (39.3%)	.189
MRA, *N* (%)	154 (23.6%)	53 (37.6%)	101 (19.7%)	**<**.**001**
Loop diuretics, *N* (%)	327 (50.2%)	97 (69.3%)	230 (45.0%)	**<**.**001**
Antiplatelet therapy, *N* (%)	177 (27.1%)	42 (29.8%)	135 (26.4%)	.426
OAC, *N* (%)	262 (40.1%)	82 (58.2%)	180 (35.2%)	**<**.**001**
Disease-modifying TTR treatment, *N* (%)^a^	287 (44.0%)	45 (31.9%)	242 (47.4%)	.**001**
Biomarkers				
Haemoglobin, g/dl	13.5 ± 5.5	13.1 ± 2.0	13.6 ± 6.1	.320
eGFR, ml/min/1.73 m²	65.4 ± 25.4	53.0 ± 24.2	69.0 ± 24.7	**<**.**001**
ALT, U/l	30.2 ± 37.0	35.8 ± 40.4	28.6 ± 35.9	.**048**
Hs troponin, ng/l^b^	42.0 (23.0, 77.3)	55.0 (36.5, 112.5)	38.0 (20.0, 65.0)	**<**.**001**
NT-proBNP, pg/ml^c^	1460.0 (284.0, 4051.3)	4051.0 (2095.5, 9433.5)	910 (144.0, 3114.5)	**<**.**001**
NAC disease stage^c^				**<**.**001**
Stage I	269 (59.6%)	30 (28.8%)	239 (68.9%)	
Stage II	103 (22.8%)	31 (29.8%)	72 (20.7%)	
Stage III	79 (17.5%)	43 (41.3%)	36 (10.4%)	
ECG parameters				
Rhythm				.**003**
Sinus rhythm	381 (59.1%)	64 (45.7%)	317 (62.8%)	
Atrial fibrillation	206 (31.9%)	62 (44.3%)	144 (28.5%)	
Junctional rhythm	4 (0.6%)	1 (0.7%)	3 (0.6%)	
Pacemaker rhythm	54 (8.4%)	13 (9.3%)	41 (8.1%)	
First degree AV block, *N* (%)	148 (35.7%)	38 (53.5%)	110 (32.1%)	**<**.**001**
QRS duration, ms	104 (90, 134)	110 (96, 142)	102 (90, 132)	.**005**
Broadened QRS, *N* (%)	203 (32.6%)	51 (38.9%)	152 (30.9%)	.081
LV hypertrophy, *N* (%)	51 (8.3%)	13 (401.2%)	38 (7.7%)	.362
Low voltage, *N* (%)	121 (19.5%)	28 (21.9%)	93 (18.9%)	.456
Pseudo-infarction, *N* (%)	136 (22.2%)	34 (26.8%)	102 (21.0%)	.166

Values are presented as mean ± SD, median (IQR) or *N* (%). Bold *P* values indicate significant *P* values (<.05).

ACE-I, angiotensin converting enzyme inhibitor; ALT, alanine aminotransferase; ARB, angiotensin receptor blocker; AV, atrioventricular; BMI, body mass index; eGFR, estimated glomerular filtration rate; Hs, high sensitivity; LV, left ventricular; MRA, mineralocorticoid receptor antagonist; NAC, National Amyloidosis Centre; NT-proBNP, N-terminal pro-B-type natriuretic peptide; NYHA, New York Heart Association; OAC, oral anticoagulant therapy.

^a^Disease-modifying treatment: 97% tafamidis, 3% TTR silencers.

^b^Available in 298/654 patients (46%).

^c^Available in 451/654 patients (69%).

**Table 2:** Baseline echocardiographic characteristics of the study population, further divided according to the occurrence of heart failure hospitalization

Variable	Overall (N = 654)	With heart failure hospitalization (N = 141)	Without heart failure hospitalization (N = 513)	*P* value
LVEDD, mm	44.8 ± 6.6	44.7 ± 7.5	44.8 ± 6.3	.968
LVESD, mm	30.9 ± 7.2	32.2 ± 8.3	30.5 ± 6.7	.**015**
LVEDV, ml	100.0 ± 33.9	98.4 ± 38.5	100.5 ± 32.5	.589
LVESV, ml	50.4 ± 24.6	55.1 ± 28.1	49.0 ± 23.4	.**032**
SV index, ml/m²	27.4 ± 10.4	24.1 ± 12.2	28.4 ± 9.6	**<**.**001**
IVSd, mm	15.3 ± 4.3	16.5 ± 4.1	15.0 ± 4.4	**<**.**001**
LV mass index, mg/m²	151.6 ± 61.0	171.0 ± 65.9	146.1 ± 58.5	**<**.**001**
LVEF, %	51.8 ± 12.3	48.2 ± 13.0	53.0 ± 11.9	**<**.**001**
LV GLS, %^a^	13.3 ± 4.9	10.9 ± 4.2	13.9 ± 4.8	**<**.**001**
LA diameter, mm	44.6 ± 8.2	47.2 ± 8.0	43.8 ± 8.1	**<**.**001**
*E*/*e*′	15.9 (10.3, 22.5)	19.0 (14.0, 27.0)	14.5 (9.0, 21.0)	**<**.**001**
TAPSE, mm	18.3 ± 5.4	16.4 ± 5.5	18.9 ± 5.3	**<**.**001**
Significant valvular lesion, *N* (%)	244 (41.4%)	80 (62.5%)	164 (35.5%)	**<**.**001**
Pericardial effusion, *N* (%)	94 (16.0%)	26 (20.5%)	68 (14.7%)	.117

Values are presented as mean ± SD, median (IQR) or *n* (%). Bold *P* values indicate significant *P* values (<.05).

IVSd, interventricular septum thickness in diastole; LA, left atrial; LVEDD, left ventricular end-diastolic diameter; LVEDV, left ventricular end-diastolic volume; LVEF, left ventricular ejection fraction; LVESD, left ventricular end-systolic diameter; LVESV, left ventricular end-systolic volume; LV GLS, left ventricular global longitudinal strain; SV, stroke volume; TAPSE, tricuspid annular plane systolic excursion.

^a^Available in 404/654 patients (62%).

### Patient outcomes

During a median follow-up of 24 months, 170 (26%) patients died. Cause of death and other cardiovascular events are reported in Supplementary Table S2.

**Figure 1:**
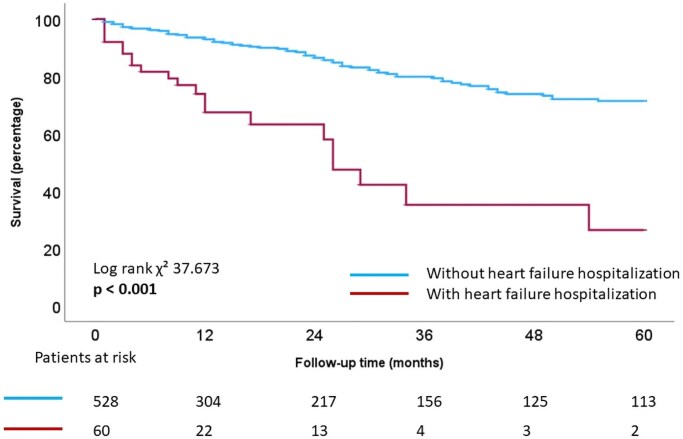
Landmark analysis for all-cause mortality for the 1-year time point. Day 0 in the landmark analysis is day 366 of the follow-up

The landmark analysis for all-cause mortality was performed at the 1-year time point (*Figure 1*). In the first year of follow-up, 60 (9%) patients had a first HFH. Sixty six (10%) patients died during the first year of follow-up and thus were excluded from the landmark analysis. The log-rank test for the landmark analysis at 1 year showed significantly worse outcomes for patients who experienced a HFH (log-rank χ² 37.673, *P* < .001).

### Association of the occurrence of a heart failure hospitalization with long-term mortality

**Figure 2:**
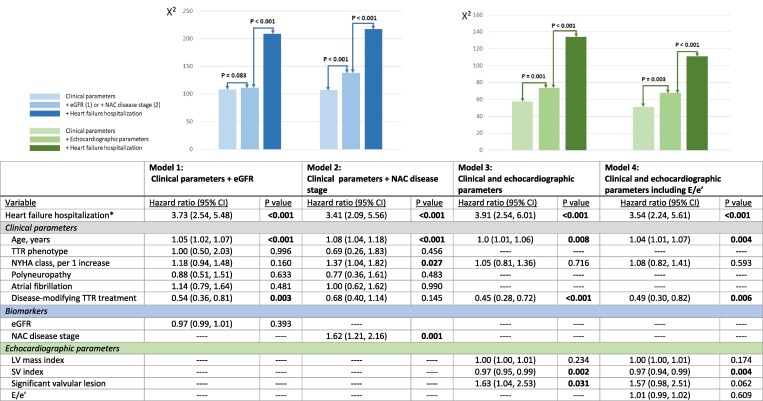
Multivariable Cox regression models and likelihood ratio test for the association with all-cause mortality. Bold *P* values indicate significant *P* values (<.05). *Heart failure hospitalization was entered as a time-dependent covariate. CI, confidence interval; TTR, transthyretin amyloid; eGFR, estimated glomerular filtration rate; NAC, National Amyloidosis Centre; NYHA, New York Heart Association; SV, stroke volume

*Table 3* shows the univariable Cox regression analysis for the association of clinical and echocardiographic variables with all-cause mortality, while *Figure 2* displays the different multivariable Cox regression analysis models. In univariable analysis, occurrence of a HFH, entered as a time-dependent covariate, was significantly associated with all-cause mortality (hazard ratio (HR) 7.71, 95% CI 5.50, 10.82; *P* < .001), as also several clinical and echocardiographic variables. Per NAC disease stage, univariable analysis showed a HR for the occurrence of a HFH of 15.50 (95% CI 7.09, 33.64; *P* < .001) in NAC disease stage I, HR 6.47 (95% CI 2.83, 14.79; *P* < .001) in NAC disease stage II and HR 3.96 (1.78, 8.72; *P* < .001) in NAC disease stage III.

**Table 3:** Univariable Cox regression analysis for the association of covariates with all-cause mortality

Variable	Wald statistic	Hazard ratio (95% CI)	*P* value
Heart Failure hospitalization (time-dependent covariate)	139.96	7.71 (5.450, 10.82)	**<**.**001**
*Clinical parameters*			
Age, years	81.79	1.08 (1.06, 1.10)	**<**.**001**
Male sex, *N* (%)	3.39	0.71 (0.50, 1.02)	.065
TTR phenotype, *N* (%)	61.81	8.59 (5.02, 14.68)	**<**.**001**
BMI, kg/m²	0.16	0.99 (0.96, 1.03)	.690
NYHA class, per 1 increase	64.15	2.14 (1.78, 2.58)	**<**.**001**
Arterial hypertension, *N* (%)	9.29	1.65 (1.20, 2.28)	.**002**
Dyslipidaemia, *N* (%)	9.27	1.65 (1.20, 2.28)	.**002**
Diabetes mellitus, *N* (%)	8.87	1.81 (1.23, 2.68)	.**003**
Current smoker, *N* (%)	2.00	0.49 (0.18, 1.32)	.157
Coronary artery disease, *N* (%)	20.18	2.20 (1.56, 3.11)	**<**.**001**
Atrial fibrillation, *N* (%)	30.41	2.53 (1.82, 3.52)	**<**.**001**
Previous stroke, *N* (%)	**24.27**	**2.88 (1.89, 4.39)**	**<**.**001**
Asthma or chronic obstructive pulmonary disease, *N* (%)	**16.59**	**3.04 (1.78, 5.19)**	**<**.**001**
Carpal tunnel syndrome, *N* (%)	**0.039**	**0.97 (0.68, 1.37)**	.**843**
Polyneuropathy, *N* (%)	**31.25**	**0.28 (0.18, 0.44)**	**<**.**001**
Autonomic dysfunction, *N* (%)	**11.86**	**0.48 (0.31, 0.73)**	**<**.**001**
Lumbar spinal stenosis, *N* (%)	**0.05**	**1.05 (0.68, 1.64)**	.**817**
Beta blocker, *N* (%)	15.07	1.89 (1.37, 2.61)	**<**.**001**
ACE-i/ARB, *N* (%)	3.59	1.36 (0.99, 1.88)	.058
MRA, *N* (%)	21.96	2.26 (1.61, 3.17)	**<**.**001**
Loop diuretics, *N* (%)	28.92	2.52 (1.80, 3.53)	**<**.**001**
Antiplatelet therapy, *N* (%)	2.57	1.33 (0.94, 1.88)	.109
OAC, *N* (%)	14.71	1.88 (1.36, 2.60)	**<**.**001**
Disease-modifying TTR treatment, *N* (%)	36.41	0.31 (0.21, 0.45)	**<**.**001**
*Biomarkers*			
Haemoglobine, g/dl	27.07	0.79 (0.73, 0.87)	**<**.**001**
eGFR, ml/min/1,73 m²	80.29	0.97 (0.96, 0.98)	**<**.**001**
ALT, U/l	2.71	1.00 (1.00, 1.01)	.100
Hs troponine, ng/l	16.40	1.00 (1.00, 1.00)	**<**.**001**
NT-proBNP, pg/ml, per 100 increase	89.56	1.01 (1.01, 1.01)	**<**.**001**
NAC disease stage	95.55	3.65 (2.82, 4.73)	**<**.**001**
*ECG parameters*			
First degree AV block, *N* (%)	8.26	2.01 (1.25, 3.23)	.**004**
QRS duration, ms	10.75	1.01 (1.00, 1.01)	.**001**
Broadened QRS, *N* (%)	10.75	1.76 (1.26, 2.47)	.**001**
LVH, *N* (%)	0.99	1.32 (0.76, 2.30)	.320
Low voltage, *N* (%)	0.61	1.18 (0.78, 1.77)	.436
Pseudo-infarction, *N* (%)	0.58	1.16 (0.79, 1.70)	.447
*Echocardiographic parameters*			
LVEDD, mm	12.36	0.95 (0.93, 0.98)	**<**.**001**
LVESD, mm	1.34	1.02 (0.99, 1.04)	.247
IVSd, mm	40.47	1.11 (1.08, 1.15)	**<**.**001**
LVEDV, ml	8.22	0.99 (0.99, 1.00)	.**004**
LVESV, ml	0.23	1.00 (0.99, 1.01)	.632
SV index	23.72	0.95 (0.93, 0.97)	**<**.**001**
LVEF, %	35.46	0.96 (0.95, 0.98)	**<**.**001**
LV GLS, %	58.36	0.83 (0.80, 0.87)	**<**.**001**
LV mass index, mg/m²	41.19	1.01 (1.01, 1.01)	**<**.**001**
LA diameter, mm	28.05	1.05 (1.03, 1.07)	**<**.**001**
*E*/*e*′	26.75	1.03 (1.02, 1.05)	**<**.**001**
TAPSE, mm	36.67	0.90 (0.87, 0.93)	**<**.**001**
Significant valvular lesion, *N* (%)	52.30	3.63 (2.56, 5.15)	**<**.**001**
Pericardial effusion, *N* (%)	9.34	1.90 (1.26, 2.87)	.**002**

Bold *P* values indicate significant *P* values (<.05).

ACE-I, angiotensin converting enzyme inhibitor; ALT, alanine aminotransferase; ARB, angiotensin receptor blocker; AV, atrioventricular; BMI, body mass index; eGFR, estimated glomerular filtration rate; Hs, high sensitivity; IVSd, interventricular septum thickness in diastole; LA, left atrial; LV, left ventricular; LVEDD, left ventricular end-diastolic diameter; LVEDV, left ventricular end-diastolic volume; LVEF, left ventricular ejection fraction; LVESD, left ventricular end-systolic diameter; LVESV, left ventricular end-systolic volume; LV GLS, left ventricular global longitudinal strain; MRA, mineralocorticoid receptor antagonist; NAC, National Amyloidosis Centre; NT-proBNP, N-terminal pro-B-type natriuretic peptide; NYHA, New York Heart Association; OAC, oral anticoagulant therapy; SV, stroke volume; TAPSE, tricuspid annular plane systolic excursion.

In multivariable analysis, occurrence of HFH remained significantly associated with outcome in all models, after adjusting for clinical parameters and biomarkers (Models 1 and 2) and after adjusting for clinical and echocardiographic parameters (Models 3 and 4). Specifically, in Models 1 and 2 together with the HFH, age, disease-modifying TTR treatment (Model 1) and New York Heart Association class (Model 2) remained associated with increased risk of all-cause mortality; these variables were included in Models 3 and 4. While NAC disease stage and HFH were independently associated with all-cause mortality (Model 2), eGFR was not (Model 1). Stroke volume index (Models 3 and 4) and the presence of a significant valvular lesion (Model 3) were the echocardiographic parameters that remained independently associated with an increased risk of all-cause mortality. Furthermore, the likelihood ratio test showed a significant *X*² increase when adding the occurrence of a HFH on top of all four models (*P* < .001 for all models). Finally, an additional multivariable model was constructed to adjust for LV global longitudinal strain, instead of stroke volume index as marker of LV systolic function (Supplementary Table S3), and *E*/*e*′. This confirmed the independent prognostic value of the occurrence of HFH.

In subgroup analysis, the association of a HFH with all-cause mortality was confirmed in univariable analysis in patients with disease-modifying TTR treatment (HR 8.83; 95% CI 4.25, 18.35; *P* < .001) and without disease-modifying TTR treatment (HR 6.88; 95% CI 4.76, 9.94; *P* < .001). In both subgroups, similar multivariable Cox regression models were constructed, adjusting for age and biomarkers (Model 1: eGFR and Model 2: NAC) and age and echocardiographic markers (Model 3: LV mass index and Model 4: SV index). These models confirmed the independent association of a HFH with all-cause mortality in both subgroups (Supplementary Table S4). Conversely, the HR for all-cause mortality for disease-modifying TTR treatment was calculated in patients with and without HFH. In patients with HFH (*N* = 141), the HR for disease-modifying treatment was 0.52 (95% CI 0.30, 0.91; *P* = .021) while in patients without HFH (*N* = 513), the HR was 0.27 (95% 0.16, 0.46; *P* < .001).

## Discussion

In this retrospective, multicentre analysis, the main findings can be summarized as follows: (i) Heart failure hospitalization is common in TTR-CA and occurred in 22% of the study population over a median follow-up of 24 months, (ii) Patients with HFH were older, more symptomatic, had more co-morbidities and showed signs of more advanced disease according to NAC disease stage and echocardiography, (iii) Patients who experienced HFH in the first year of follow-up had significantly worse outcome, (iv) The occurrence of HFH was independently associated with increased all-cause mortality and provided incremental value on top of all other risk models, (v) The results were confirmed in subgroup analysis evaluating patients with and without disease-modifying TTR treatment.

### The occurrence of heart failure hospitalization in transthyretin cardiac amyloidosis

Due to the increased disease awareness and non-invasive diagnostic imaging options, the prevalence of TTR-CA is now considered to be ∼20% in patients with cardiomyopathies with hypertrophic phenotype older than 50 year,^20–22^ 6%–13% in patients with HF and preserved ejection fraction,^23,24^ and 16% in patients with severe aortic stenosis treated with transcatheter aortic valve replacement.^25^ While the natural prognosis of TTR-CA is poor,^26^ disease-modifying TTR treatments have recently demonstrated to improve outcome.^1,2^ Still, HFH remains a frequent adverse event in these patients. In the Transthyretin Amyloidosis Cardiomyopathy Clinical Trial, 138 (52%) of 264 patients treated with the TTR stabilizer tafamidis had a cardiovascular related hospitalization as compared with 107 (61%) of 177 patients in the placebo group, during a median follow-up of 33 months.^1^ In the Helios B trial, 112 (34%) of 326 patients treated with the TTR RNA silencer vutrisiran had a recurrent cardiovascular event, defined as a cardiovascular related hospitalization or urgent HF visit, as compared with 133 (41%) of 329 patients in the placebo group during a median follow-up of 36 months.^2^ The high incidence of cardiovascular related hospitalizations in the treatment arm of these trials confirms that the occurrence of HFH remains an important issue, even with novel treatment options available.

In the current study, 141 (22%) of 654 patients experienced a HFH during a median follow-up of 24 months with a 44% baseline treatment with disease-modifying TTR treatment. The lower incidence in the current study population as compared with the randomized clinical trials, can be explained by the stricter definition of a HFH compared with the cardiovascular related hospitalizations evaluated in the trials as well as a shorter follow-up time. Conversely, in an observational study by Porcari *et al*.^27^, which investigated the influence of sodium-glucose co-transporter 2 (SGLT2)-inhibition on outcome in TTR-CA patients, a HFH occurred in 26 (12%) of 220 patients treated with a SGLT2-inhibitor as compared with 74 (34%) of 220 matched control patients during a median follow-up of 28 months. Of note, 21% of these patients received disease-modifying TTR treatment. The lower incidence of HFH might be related to the use of SGLT2-inhibitors, but prospective trials should further investigate these findings.

### The prognostic impact of heart failure hospitalization in transthyretin cardiac amyloidosis

The occurrence of a HFH has proven to significantly increase the risk of all-cause mortality in patients with HF.^11,12^ Neurohormonal activation and increased LV filling pressures during HFH induce myocardial injury which contribute to the worsened prognosis after HFH.^28^ In patients with TTR-CA, *post hoc* analyses of the Apollo B trial and Helios B trial recently evaluated the impact on all-cause mortality of a worsening outpatient HF (defined as an initiation or sustained increase in loop diuretic dose for at least seven days),^29,30^ which was indeed associated with increased mortality in both analyses.^29,30^ Whether this also applies to HFH in a real-world patients with TTR-CA, remained unclear. Ladefoged *et al*.^31^ investigated a small patient cohort with wild-type TTR-CA without disease-modifying TTR treatment, and found an occurrence of 51% of HFH during a median follow-up of 23 months. They demonstrated worse outcome in patients after HFH as compared with patients without HFH in a landmark analysis at 1 year but without correcting for other prognostic factors. Current analysis supports these findings in a larger population with both hereditary and wild-type TTR-CA and 44% of baseline treatment with disease-modifying TTR treatment, reflecting a real-world TTR-CA population. Furthermore, the current study showed additive prognostic value of a HFH on top of four different risk models including biomarkers (*Figure 2*, Models 1 and 2) and including echocardiographic markers (*Figure 2*, Models 3 and 4).

### Clinical implications

Currently in clinical practice, risk stratification of TTR-CA patients is primarily based on biomarkers^4,5^ and echocardiographic assessment.^6^ The incremental prognostic value of a HFH to these established risk factors shown in the current study, should encourage clinicians to reassess the risk profile of patients after the occurrence of a HFH. Also, these findings should stimulate the identification of patients at risk before HFH occurs, in which early HF treatment and disease-modifying TTR treatment could potentially alter the prognosis. Conventional HF therapies are currently not widely prescribed in TTR-CA.^32^ Based on small-scale studies performed in the era before disease-modifying TTR treatment, the use of conventional HF therapies like angiotensin converting enzyme-inhibitors/angiotensin receptor blockers and beta-blockers, is currently not indicated in patients with TTR-CA who develop signs of heart failure.^13,33,34^ Nevertheless, in a large retrospective analysis, mineralocorticoid receptor antagonists were associated with reduced risk of mortality in the overall study population with TTR-CA while low-dose beta-blockers were associated with reduced risk of mortality in patients with reduced LV ejection fraction <40%.^32^ However, 19% of these patients received disease-modifying TTR treatment.^32^ Furthermore, the use of SGLT2-inhibitors in TTR-CA seems promising, but needs further evaluation in prospective studies.^27,35^ Whether implementation of conventional guideline-directed HF therapies in TTR-CA could reduce the occurrence of HFH is still unknown, especially considering the increasing use of disease-modifying TTR treatment. In the current study, the subgroup analysis in patients with and without disease-modifying TTR treatment indicates that a HFH is still associated with worse outcome in patients receiving disease-modifying TTR treatment, although lower than in the ones not treated.

Although currently the clinical goals are focused on early diagnosis of TTR-CA and timely treatment, initiation or continuation of disease-modifying TTR treatment after HFH might not be futile and could still be considered. The exact impact of the (combination of) disease-modifying TTR treatment and HF therapies on the occurrence of HFH should be input for further prospective studies.

Finally, the occurrence of a HFH should be taken into account in clinical trials either to define the disease stage and risk profile of these patients, or as important endpoint.

### Study limitations

This multicentre, international study has a retrospective design and should be interpreted in this context. No data were available on the characteristics of the patients at the time of the HFH nor the precipitating causes for the HFH nor the specific treatment during HFH, and this might have differed among the centres. Nevertheless, the inclusion of different multinational centres in this study population allows for a broad representation of the real-world TTR-CA patient population worldwide.

## Conclusion

In this real-life multicentre cohort with TTR-CA, HFH was a frequent complication and associated with significantly reduced survival. Occurrence of HFH was independently associated with all-cause mortality on top of established risk models. These findings should stimulate clinicians to reassess the risk of patients with TTR-CA after HFH, diagnose patients with TTR-CA before HFH occurs and initiate disease-modifying TTR treatment and perhaps HF treatment to potentially alter the prognosis of the patients.

## Supplementary Material

xvaf013_Supplementary_Data

## Declarations

### Data Availability

No data were generated or analysed for this manuscript.

### Pre-registered Clinical Trial Number

None supplied.

## References

[1] Maurer MS, Schwartz JH, Gundapaneni B, Elliott PM, Merlini G, Waddington-Cruz M, et al Tafamidis treatment for patients with transthyretin amyloid cardiomyopathy. N Engl J Med 2018;379:1007–16. 10.1056/NEJMoa1805689 30145929

[2] Fontana M, Berk JL, Gillmore JD, Witteles RM, Grogan M, Drachman B, et al Vutrisiran in patients with transthyretin amyloidosis with cardiomyopathy. N Engl J Med 2024;392:33–44. 10.1056/NEJMoa2409134 39213194

[3] Ruberg FL, Grogan M, Hanna M, Kelly JW, Maurer MS. Transthyretin amyloid cardiomyopathy: JACC state-of-the-art review. J Am Coll Cardiol 2019;73:2872–91. 10.1016/j.jacc.2019.04.003 31171094 PMC6724183

[4] Grogan M, Scott CG, Kyle RA, Zeldenrust SR, Gertz MA, Lin G, et al Natural history of wild-type transthyretin cardiac amyloidosis and risk stratification using a novel staging system. J Am Coll Cardiol 2016;68:1014–20. 10.1016/j.jacc.2016.06.033 27585505

[5] Gillmore JD, Damy T, Fontana M, Hutchinson M, Lachmann HJ, Martinez-Naharro A, et al A new staging system for cardiac transthyretin amyloidosis. Eur Heart J 2018;39:2799–806. 10.1093/eurheartj/ehx589 29048471

[6] Chacko L, Martone R, Bandera F, Lane T, Martinez-Naharro A, Boldrini M, et al Echocardiographic phenotype and prognosis in transthyretin cardiac amyloidosis. Eur Heart J 2020;41:1439–47. 10.1093/eurheartj/ehz905 31950987

[7] McMurray JJ, Packer M, Desai AS, Gong J, Lefkowitz MP, Rizkala AR, et al Angiotensin-neprilysin inhibition versus enalapril in heart failure. N Engl J Med 2014;371:993–1004. 10.1056/NEJMoa1409077 25176015

[8] Anker SD, Butler J, Filippatos G, Ferreira JP, Bocchi E, Böhm M, et al Empagliflozin in heart failure with a preserved ejection fraction. N Engl J Med 2021;385:1451–61. 10.1056/NEJMoa2107038 34449189

[9] Packer M, Anker SD, Butler J, Filippatos G, Pocock SJ, Carson P, et al Cardiovascular and renal outcomes with empagliflozin in heart failure. N Engl J Med 2020;383:1413–24. 10.1056/NEJMoa2022190 32865377

[10] Armstrong PW, Pieske B, Anstrom KJ, Ezekowitz J, Hernandez AF, Butler J, et al Vericiguat in patients with heart failure and reduced ejection fraction. N Engl J Med 2020;382:1883–93. 10.1056/NEJMoa1915928 32222134

[11] Solomon SD, Dobson J, Pocock S, Skali H, McMurray JJ, Granger CB, et al Influence of nonfatal hospitalization for heart failure on subsequent mortality in patients with chronic heart failure. Circulation 2007;116:1482–7. 10.1161/CIRCULATIONAHA.107.696906 17724259

[12] Carson PE, Anand IS, Win S, Rector T, Haass M, Lopez-Sendon J, et al The hospitalization burden and post-hospitalization mortality risk in heart failure with preserved ejection fraction: results from the I-PRESERVE trial (irbesartan in heart failure and preserved ejection fraction). JACC Heart Fail 2015;3:429–41. 10.1016/j.jchf.2014.12.017 25982110

[13] Garcia-Pavia P, Rapezzi C, Adler Y, Arad M, Basso C, Brucato A, et al Diagnosis and treatment of cardiac amyloidosis. A position statement of the European Society of Cardiology Working Group on Myocardial and Pericardial Diseases. Eur J Heart Fail 2021;23:512–26. 10.1002/ejhf.2140 33826207

[14] Lang RM, Badano LP, Mor-Avi V, Afilalo J, Armstrong A, Ernande L, et al Recommendations for cardiac chamber quantification by echocardiography in adults: an update from the American Society of Echocardiography and the European Association of Cardiovascular Imaging. J Am Soc Echocardiogr 2015;28:1–39.e14. 10.1016/j.echo.2014.10.003 25559473

[15] Devereux RB, Alonso DR, Lutas EM, Gottlieb GJ, Campo E, Sachs I, et al Echocardiographic assessment of left ventricular hypertrophy: comparison to necropsy findings. Am J Cardiol 1986;57:450–8. 10.1016/0002-9149(86)90771-X 2936235

[16] Voigt JU, Pedrizzetti G, Lysyansky P, Marwick TH, Houle H, Baumann R, et al Definitions for a common standard for 2D speckle tracking echocardiography: consensus document of the EACVI/ASE/Industry Task Force to standardize deformation imaging. Eur Heart J Cardiovasc Imaging 2015;16:1–11. 10.1093/ehjci/jeu184 25525063

[17] Nagueh SF, Smiseth OA, Appleton CP, Byrd BF 3rd, Dokainish H, Edvardsen T, et al Recommendations for the evaluation of left ventricular diastolic function by echocardiography: an update from the American Society of Echocardiography and the European Association of Cardiovascular Imaging. J Am Soc Echocardiogr 2016;29:277–314. 10.1016/j.echo.2016.01.011 27037982

[18] Lancellotti P, Tribouilloy C, Hagendorff A, Popescu BA, Edvardsen T, Pierard LA, et al Recommendations for the echocardiographic assessment of native valvular regurgitation: an executive summary from the European Association of Cardiovascular Imaging. Eur Heart J Cardiovasc Imaging 2013;14:611–44. 10.1093/ehjci/jet105 23733442

[19] Zoghbi WA, Adams D, Bonow RO, Enriquez-Sarano M, Foster E, Grayburn PA, et al Recommendations for noninvasive evaluation of native valvular regurgitation: a report from the American Society of Echocardiography Developed in Collaboration with the Society for Cardiovascular Magnetic Resonance. J Am Soc Echocardiogr 2017;30:303–71. 10.1016/j.echo.2017.01.007 28314623

[20] Garcia-Pavia P, Damy T, Piriou N, Barriales-Villa R, Cappelli F, Bahus C, et al Prevalence and characteristics of transthyretin amyloid cardiomyopathy in hypertrophic cardiomyopathy. ESC Heart Fail 2024;11:4314–24. 10.1002/ehf2.14971 39210606 PMC11631301

[21] Bakalakos A, Monda E, Elliott PM. The diagnostic and therapeutic implications of phenocopies and mimics of hypertrophic cardiomyopathy. Can J Cardiol 2024;40:754–65. 10.1016/j.cjca.2024.02.025 38447917

[22] Angelini F, Bocchino PP, Dusi V, Pidello S, De Ferrari GM, Raineri C. From thick walls to clear answers: approaches to diagnosing hypertrophic cardiomyopathy and its mimics. Eur Heart J Suppl 2025;27:i40–i6. 10.1093/eurheartjsupp/suae099 39980777 PMC11836689

[23] González-López E, Gallego-Delgado M, Guzzo-Merello G, de Haro-Del Moral FJ, Cobo-Marcos M, Robles C, et al Wild-type transthyretin amyloidosis as a cause of heart failure with preserved ejection fraction. Eur Heart J 2015;36:2585–94. 10.1093/eurheartj/ehv338 26224076

[24] AbouEzzeddine OF, Davies DR, Scott CG, Fayyaz AU, Askew JW, McKie PM, et al Prevalence of transthyretin amyloid cardiomyopathy in heart failure with preserved ejection fraction. JAMA Cardiol 2021;6:1267–74. 10.1001/jamacardio.2021.3070 34431962 PMC8387947

[25] Castaño A, Narotsky DL, Hamid N, Khalique OK, Morgenstern R, DeLuca A, et al Unveiling transthyretin cardiac amyloidosis and its predictors among elderly patients with severe aortic stenosis undergoing transcatheter aortic valve replacement. Eur Heart J 2017;38:2879–87. 10.1093/eurheartj/ehx350 29019612 PMC5837725

[26] Nativi-Nicolau J, Judge DP, Hoffman JE, Gundapaneni B, Keohane D, Sultan MB, et al Natural history and progression of transthyretin amyloid cardiomyopathy: insights from ATTR-ACT. ESC Heart Fail 2021;8:3875–84. 10.1002/ehf2.13541 34432383 PMC8497209

[27] Porcari A, Cappelli F, Nitsche C, Tomasoni D, Sinigiani G, Longhi S, et al SGLT2 inhibitor therapy in patients with transthyretin amyloid cardiomyopathy. J Am Coll Cardiol 2024;83:2411–22. 10.1016/j.jacc.2024.03.429 38866445

[28] Gheorghiade M, De Luca L, Fonarow GC, Filippatos G, Metra M, Francis GS. Pathophysiologic targets in the early phase of acute heart failure syndromes. Am J Cardiol 2005;96:11g–7g. 10.1016/j.amjcard.2005.07.016 16196154

[29] Fontana M, Maurer MS, Gillmore JD, Bender S, Jay PY, Solomon SD. Worsening of heart failure in outpatients with transthyretin amyloidosis and cardiomyopathy in the APOLLO-B trial. J Am Coll Cardiol 2025;85:744–52. 10.1016/j.jacc.2024.10.097 39846936

[30] Fontana M, Maurer MS, Gillmore JD, Bender S, Aldinc E, Eraly SA, et al Outpatient worsening heart failure in patients with transthyretin amyloidosis with cardiomyopathy in the HELIOS-B trial. J Am Coll Cardiol 2025;85:753–61. 10.1016/j.jacc.2024.11.015 39566871

[31] Ladefoged BT, Dybro A, Dahl Pedersen AL, Rasmussen TB, Vase H, Clemmensen TS, et al Incidence and predictors of worsening heart failure in patients with wild-type transthyretin cardiac amyloidosis. ESC Heart Fail 2022;9:2978–87. 10.1002/ehf2.14000 35733407 PMC9715879

[32] Ioannou A, Massa P, Patel RK, Razvi Y, Porcari A, Rauf MU, et al Conventional heart failure therapy in cardiac ATTR amyloidosis. Eur Heart J 2023;44:2893–907. 10.1093/eurheartj/ehad347 37216684 PMC10424879

[33] Aus dem Siepen F, Hein S, Bauer R, Katus HA, Kristen AV. Standard heart failure medication in cardiac transthyretin amyloidosis: useful or harmful? Amyloid 2017;24:132–3. 10.1080/13506129.2016.1272453 28434295

[34] Cheng RK, Vasbinder A, Levy WC, Goyal P, Griffin JM, Leedy DJ, et al Lack of association between neurohormonal blockade and survival in transthyretin cardiac amyloidosis. J Am Heart Assoc 2021;10:e022859. 10.1161/JAHA.121.022859 34729989 PMC9075255

[35] Laborante R, Elia S, Savarese G, Patti G, D'Amario D. Tolerability and efficacy of sodium-glucose co-transporter 2 inhibitors in patients with cardiac amyloidosis: a meta-analysis of observational studies. Eur Heart J Cardiovasc Pharmacother 2025;11:356–64. 10.1093/ehjcvp/pvaf033 40380981 PMC12231128

