# Supplementary material for: Prognostic value of heart failure hospitalization in transthyretin cardiac amyloidosis: an international cohort study
Source: ESC Heart Fail. 2026 Jan 8;13(1):xvaf013. doi: 10.1093/eschf/xvaf013 (PMC13108267; doi:10.1093/eschf/xvaf013)
Supplement: xvaf013_Supplementary_Data [file xvaf013_supplementary_data.docx]

**Supplemental materials**

**Supplemental Table 1. List of participating centers**

| **Center** |
| --- |
| Leiden University Medical Center, Leiden, The Netherlands |
| Sint Jan Hospital Brugge, Bruges, Belgium |
| Vrije Universiteit Brussel, Brussels, Belgium |
| Universitair Ziekenhuis Antwerpen, Antwerp, Belgium |
| AZ Maria Middelares, Gent, Belgium |
| Ziekenhuis Oost-Limburg, Genk, Belgium |
| University of Rennes, CHU Rennes, France |
| Centro Hospitalar Universitario de Lisboa Norte, Lisbon, Portugal |
| University of Medicine and Pharmacy ‘Carol Davila’, Bucharest, Romania |
| Rabin Medical Centre, Petah Tikva, Israel |
| National University Heart Center, Singapore, Singapore |
| University of Hong Kong – Shenzhen Hospital, Hong Kong, China |
| Kitasato University Hospital, Sagamihara, Japan |
| Tokyo Medical and Dental University, Tokyo, Japan |
| Saint Marianna University School of Medicine, Kawasaki, Japan |
| Universidad de Buenos Aires, Buenos Aires, Argentina |
| University of New South Wales, Sydney, Australia |

**Supplemental Table 2. Cardiovascular events during follow-up**

| Variable | Overall  (N = 654) | With Heart Failure Hospitalization  (N = 141) | Without Heart Failure Hospitalization  (N = 513) | p value |
| --- | --- | --- | --- | --- |
| Deaths   - Cardiac death - Non-cardiac death - Unknown cause of death | 170 (26%)  53 (37%)  44 (30%)  46 (32%) | 81 (57.4%)  33 (52.4%)  16 (25.4%)  14 (22.2%) | 89 (17.3%)  20 (25%)  28 (35%)  32 (40%) | **<0.001** |
| De novo atrial fibrillation, N (%) (excluding patients with AF at baseline N = 261) | 129 (20.1%) | 56 (40.6%) | 73 (14.5%) | **<0.001** |
| Stroke, N (%) | 20 (3.1%) | 9 (6.6%) | 11 (2.1%) | **0.008** |
| Pacemaker implantation, N (%) | 88 (13.6%) | 20 (14.6%) | 68 (13.3%) | 0.689 |
| Ventricular arrhythmias, N (%) | 16 (2.6%) | 9 (6.6%) | 7 (1.5%) | **<0.001** |

Bold p values indicate significant p values (<0.05).

**Supplemental Table 3. Additional multivariable model (including LV GLS and E/e) for the association with all-cause mortality**

|  | Additional model 2: including E/e’ |  |
| --- | --- | --- |
| Variable | Hazard ratio (95% CI) | P value |
| Heart failure hospitalization* | 4.43 (2.65, 7.39) | **<0.001** |
| Age, years | 1.06 (1.03, 1.10) | **<0.001** |
| NYHA class, per 1 increase | 1.31 (0.93, 1.83) | 0.118 |
| Disease-modifying TTR treatment | 0.59 (0.34, 1.02) | 0.059 |
| LV mass index | 1.01 (1.00, 1.01) | 0.529 |
| LV GLS | 0.90 (0.84, 0.98) | **0.010** |
| Significant valvular lesion | 1.78 (1.00, 3.16) | 0.051 |
| E/e’ | 1.00 (0.97, 1.02) | 0.644 |

Bold p values indicate significant p values (<0.05).
*Heart failure hospitalization was entered as a time dependent covariate
Abbreviations: TTR = transthyretin amyloid; CI = confidence interval; LV = left ventricular; LV GLS = left ventricular global longitudinal strain; NYHA = New York Heart Association.

**Supplemental Table 4. Multivariable Cox regression analysis for the association of variables with all-cause mortality in patient groups treated with or without disease-modifying TTR treatment**

| With disease modifying TTR treatment | | | | | | | | |
| --- | --- | --- | --- | --- | --- | --- | --- | --- |
|  | Model 1 | | Model 2 | | Model 3 | | Model 4 | |
| Variable | Hazard ratio (95% CI) | P value | Hazard ratio (95% CI) | P value | Hazard ratio (95% CI) | P value | Hazard ratio (95% CI) | P value |
| Heart failure hospitalization* | 5.28 (2.49,11.20) | **<0.001** | 4.61 (1.64, 12.99) | **0.004** | 5.12 (2.34, 11.20) | **<0.001** | 4.18 (1.71, 10.26) | **0.002** |
| *Clinical parameters* | | | | | | | | |
| Age, years | 1.06 (1.02, 1.09) | **<0.001** | 1.05 (1.01, 1.09) | **0.021** | 1.06 (1.03, 1.09) | **<0.001** | 1.04 (1.01, 1.08) | **0.023** |
| *Biomarkers* | | | | | | | | |
| eGFR | 1.00 (0.98, 1.02) | 0.977 | ---- |  | ---- | ---- | ---- | ---- |
| NAC disease stage | ---- | ---- | 2.73 (1.41, 5.29) | **0.003** | ---- | ---- | ---- | ---- |
| *Echocardiographic parameters* | | | | | | | | |
| LV mass index | ---- | ---- | ---- | ---- | 1.00 (0.99, 1.01) | 0.678 | ---- | ---- |
| SV index | ---- | ---- | ---- | ---- | ---- | ---- | 0.97 (0.93, 1.02) | 0.191 |

| Without disease modifying TTR treatment | | | | | | | | |
| --- | --- | --- | --- | --- | --- | --- | --- | --- |
| Heart failure hospitalization* | 4.13 (2.77, 6.17) | **<0.001** | 4.45 (2.59, 7.64) | **<0.001** | 4.68 (3.14, 6.96) | **<0.001** | 3.78 (2.44, 5.87) | **<0.001** |
| *Clinical parameters* | | | | | | | | |
| Age, years | 1.05 (1.03, 1.07) | **<0.001** | 1.06 (1.04, 1.10) | **<0.001** | 1.05 (1.03, 1.07) | **<0.001** | 1.05 (1.02, 1.07) | **<0.001** |
| *Biomarkers* | | | | | | | | |
| eGFR | 0.99 (0.98, 1.00) | 0.198 | ---- |  | ---- | ---- | ---- | ---- |
| NAC disease stage | ---- | ---- | 1.47 (1.08, 1.99) | **0.014** | ---- | ---- | ---- | ---- |
| *Echocardiographic parameters* | | | | | | | | |
| LV mass index | ---- | ---- | ---- | ---- | 1.00 (1.00, 1.01) | **0.039** | ---- | ---- |
| SV index | ---- | ---- | ---- | ---- | ---- | ---- | 0.96 (0.94, 0.98) | **<0.001** |

**Supplemental Figure 1. Patient selection flowchart**

**
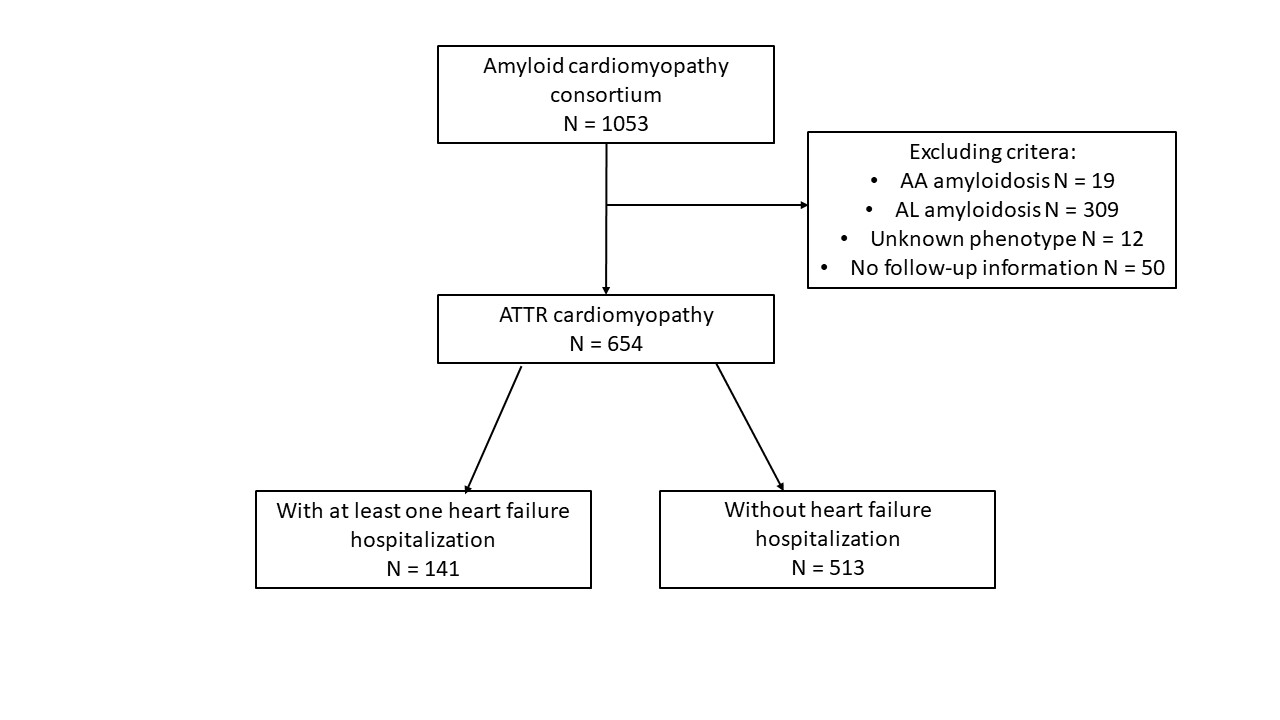
**
